# Rural women choose self-sampling over a pelvic exam for cervical cancer screening: a mixed-method study

**DOI:** 10.1007/s10552-025-02081-5

**Published:** 2025-10-27

**Authors:** Timothy C. Guetterman, Alexandra Vinson, Matthew Stack, Martha L. Alves, Elizabeth Haro, Emma Alman, Butcher, Christelle El Khoury, Melissa DeJonckheere, Diane M. Harper

**Affiliations:** 1 Department of Family Medicine, University of Michigan, 1018 Fuller Street, Ann Arbor, MI 48104 USA; 2 Genesee Health System, Flint, MI USA; 3 Department of Learning Health Sciences, University of Michigan, Ann Arbor, MI USA; 4 Department of Obstetrics and Gynecology, University of Michigan, 1018 Fuller Street, Ann Arbor, MI 48104 USA; 5 Department of Women’s and Gender Studies, University of Michigan, 1018 Fuller Street, Ann Arbor, MI 48104 USA

**Keywords:** Pelvic exam, Rural, Cervical cancer screening

## Abstract

**Background:**

Barriers to cervical cancer screening are significantly higher among US rural populations. To understand these barriers and explore potential remedies, we compare perceptions of screening exam techniques, pelvic exam vs. self-sampling, and how perceptions vary by participants’ beliefs, physician characteristics, and known barriers among under-screened rural people in Michigan, United States.

**Methods:**

Our mixed-methods study explored experiences with a vaginal self-sampling technique in comparison to the memory of the most recent pelvic exam. We developed quantitative survey questions using Health Information National Trends Survey (HINTS) modules. We created the qualitative interview guide using Likert scales and the Theoretical Domains Framework (TDF). We provided vaginal self-sampling kits (HerSwab) to participants to try this new test modality. We used descriptive statistics and t tests to analyze quantitative data. We analyzed the interview responses thematically.

**Results:**

Of the forty rural white women who shared their experiences of the two screening techniques, the pelvic exam technique had significantly worse negative ratings across all fourteen perceptions than the self-sampling technique, and the self-sampling technique had significantly higher positive ratings.

Analysis of interviews revealed four themes that elaborated survey results: 1) preference for the self-sampling technique; 2) physical and emotional discomfort with the pelvic exam technique; 3) convenience of the self-sampling technique; and 4) empowerment through self-sampling.

**Conclusions:**

The powerful negative perceptions of the pelvic exam may be why people do not participate in screening. The self-sampling cervical cancer screening technique offers a quick and easy method for screening that many prefer.

**Supplementary Information:**

The online version contains supplementary material available at 10.1007/s10552-025-02081-5.

## Introduction

Cervical cancer screening has reached about 75% of the screen-eligible US population [1]. Still, half of the cervical cancers that continue to occur are from people with a cervix who never or rarely participate in screening [2]. Cervical cancer screening with primary HPV testing is the United States Preventive Services Task Force (USPSTF) grade A recommendation for those aged 30 through 65 years [3]. The average age of diagnosis for cervical cancer is 50 years, and the incidence for those 30–44 years is less than that of those aged 45–64 years (40.7/100,000 vs. 50.2/100,000) [4]. This raises the question of whether a person’s age affects the choice of screening technique.

Rural women in the United States participate in cervical cancer screening at an average 15% lower rate than their urban counterparts [5]. They are predominantly White and have significantly later-stage cervical cancers at diagnosis with higher mortality than urban White women [6, 7]. Some research ascribes this disparity to insufficient physicians in rural areas [8–11], including the characteristics of physicians, such as their gender, religion/culture, or race. Other researchers speak of the women’s beliefs or prior experiences as drivers of pelvic exam acceptance, such as her uncomfortableness, her religious beliefs, and the gender of the physician [12–14].

While research has documented the pelvic exam as a barrier to screening, other research shows that environmental factors and lack of knowledge also contribute to low participation in screening. Environmental factors like rurality, insurance, education, employment, and a low number of health clinics in the area are barriers [9]. Knowledge barriers, such as knowing that a screen was needed, when a screen was needed, and where the screen could be performed, are some of the many elements of lacking knowledge [15].

Many studies show that self-sampling is a validated option that is non-inferior to the clinician-directed exam and that does not require a pelvic exam to accomplish cervical cancer screening [16]. Mixed methods and survey studies in the US indicate that self-sampling is feasible and acceptable to women [17–20].^.^ While concordance research between the clinician-directed exam and self-sampling in US communities is limited [16, 21, 22], it is robust internationally [23–27].

HerSwab has been approved by Health Canada and is CE-marked for medical use in Canada and Europe. At the time of the study, there were no FDA-approved devices for use in the US. The option to self-screen might change the up-to-date cervical cancer screening rate of White, rural Mid-Michigan women, whose current screening is less than 30% compared to 48.6% of rural US women [28, 29].

Our primary aim is to explore the role of pelvic exam techniques in shaping cervical cancer screening experiences in rural Michigan women. We aim to compare women’s perceptions of two screening exam techniques in asymptomatic women: the conventional pelvic exam versus the self-sampling technique experienced in this study. We examine variation in the perceptions of these exams by demographic descriptors and three barriers: environmental/knowledge barriers, physician characteristics, and influencers of women’s beliefs.

## Methods

### Overview of mixed-methods methodology

We used a convergent mixed-methods design where all participants provided quantitative and qualitative data [30]. The study design was influenced by the Theoretical Domains Framework (TDF), which applies a theory-informed approach to identify determinants of behavior [31]. The conceptual model has been used in similar settings and populations [32, 33]. Supplementary Table 1 integrates the quantitative interview questions with the qualitative methods, aligning with the TDF.

### Population -recruitment

We recruited study participants through a collaboration with a non-profit health system serving 23 rural counties in central Michigan, whose main office had 18 providers who served 9000 residents. The health system facilitated the identification of women aged 30–45 (younger) and 46–65 (older) with no record of cervical cancer screening in the past three years or longer by medical record validation. The two age groups represented women in different reproductive phases of life. The vaginal environment changes between the reproductive stage and the menopausal stage. The vagina becomes less flexible, less moist, and smaller in size, possibly creating a worse experience for self-sampling.

Interested participants were contacted via telephone to provide a study description and assess eligibility and interest in participating. Inclusion criteria were aged 30–65, having a cervix (no hysterectomy), being due or overdue for cervical cancer screening (no prior screening for at least three years), and self-identified rural residency. Exclusion criteria were self-report of current pregnancy or planning to become pregnant in the next six months, a current cancer diagnosis (not including non-melanoma skin cancer), and use of a pessary.

We mailed all interested and eligible women a written informed consent document and contact information for the study team. The University of Michigan and the MyMichigan Health System approved this study under HUM 000163301. Forty-one participants were recruited; one was excluded from analysis due to incomplete data.

### HPV self-sampling kits and surveys

We used a vaginal swab (HerSwab, Eve Medical, Toronto, ON, CA) as the collection device for this study. We provided written and pictured instructions for vaginal sampling for HPV and encouraged anyone to call the study team with any questions. Participants were told that self-sampling and clinician sampling were not different in accuracy. The self-sampling kit included a demographic survey, which participants completed before self-sampling. After self-sampling, participants completed the TDF-guided survey to help the research team understand their screening methods(s). After the women returned the kits and surveys by mail, the research staff arranged a one-on-one semi-structured phone interview. This project was not IRB-approved for clinical use of the results of the HPV testing. Still, we gave the women their results; if they were abnormal, we asked them to discuss them with their documented primary care physician.

### Quantitative survey

The social-ecological model (SEM) defines the five categories of barriers influencing cervical cancer screening [34]. These are **Individual** (intrapersonal- lack of information, knowledge, awareness, low-risk perception, fear, competing priorities, embarrassment, the discomfort of screening), **Social** (interpersonal, networks, support systems, gender relations, mistrust, association with sexually transmitted infection taboos, ‘women’s body parts are private and stigmatized’), **Cultural/Traditional/Religious** (beliefs, lack of partner support, community factors), **Health System** (institutional, organizational, having insurance, routine health care opportunities, long wait times), and **Structural** (financial constraints, lack of female-focused care, low education levels, built environment (crime, transport, etc.), national laws and policies) categories [35]. All categories influence the willingness of a woman to participate in an office pelvic exam for cervical cancer screening.

The Health Information National Trends Survey (HINTS) cycle 5, developed by the National Cancer Institute (NCI), modules of demographics, cervical cancer, cancer perceptions and knowledge, health status, patient-provider communication, and risk perceptions pre-define the barriers asked of all participants in the quantitative survey [36].

We used the TDF to develop questions about the two exam techniques. Women evaluated 14 perceptions of the pelvic exam and self-sampling technique using a five-point Likert scale where 1 was Not at All (perception) and 5 was Very (perception). The negative perceptions were time-consuming, complicated, stressful, embarrassing, vulnerable, annoying, icky/gross, painful, awkward, intrusive, and uncomfortable. The positive perceptions were empowering, easy, and quick. All women had previous experience with a pelvic exam and reported their experience with the vaginal self-sampling exam they took as part of this study.

All women rated the importance of their physician’s characteristics, including gender, race/ethnicity, and religion/culture, on a five-point Likert scale. Finally, each participant rated whether she avoided getting a pelvic exam because of her own religious or cultural reasons (1 strongly disagree to 5 strongly agree). All study participants answered the survey before the interview.

### Semi-structured interview

An interpretive description approach informed the data analysis [37]. Interpretive description aims to draw on the expertise and experience of participants to understand a problem and develop candidate solutions to address it. This approach motivated us to gather the perspectives of rural White women in Michigan; by identifying shared experiences among women in this population, we seek to inform future interventions to increase low cervical cancer screening rates. In keeping with this goal, the research team developed the interview guide to elicit past experiences of cervical cancer screening and perspectives on self-sampling techniques.

We designed the phone interview to be a brief follow-up, during which participants were asked whether they would self-collect again or in the future, whether they would like to collect the self-sample at home or a doctor’s office, whether they would like to sample their own vaginas or have the doctor take the sample, what feedback they had on the instructions and packaging provided with each kit, and whether and how they would recommend the use of a self-sampling kit to a friend/family member.

The interview guides were focused, allowing the participants to complete the interview in an average of 8.5 min. The team members, all women trained in interviewing techniques (EH, MLA, CEK, EAB), conducted the interviews. No members of the research team had a prior relationship with the participants. Interviewers explained the study goals during the interview, but interviews were not returned to participants for review and correction. Interviews were audio-recorded and transcribed to facilitate qualitative analysis. Two women did not consent to audio recording but agreed for the interviewer to take detailed notes.

## Analysis

### Inductive qualitative analysis

Four study team members (EAB, MLA, CEK, EH) developed the initial coding framework by independently familiarizing themselves with the data and reviewing a subset of interview transcripts to identify the main ideas expressed by the interviewees inductively. After this initial review, the team collaboratively discussed and defined the main ideas for the coding framework. All four study team members manually coded the interviews, and two independently coded each. We used an Excel spreadsheet to manage the data during the coding process. Team members discussed any discrepancies in code application until the team achieved consensus. After responses were coded, team members discussed common themes across interviews. Saturation is not a standard for the quality or completeness of data collection in the interpretive description tradition. Nevertheless, the interview guide was focused, and the population was relatively homogenous, which supports the discovery of repeated ideas that allow theme generation. These patterns may reflect trends in individuals’ experiences beyond the immediate study sample.

### Statistical analysis

A sample size of 20 responders in each group was estimated to provide 90% power with a type 1 error of 0.05 to detect a difference of one point on the Likert rating scale, assuming a standard deviation of one. One point is a clinically significant difference [38, 39]. We used frequencies, chi-square, and Kruskal–Wallis (or Mann–Whitney U testing) testing of the demographic variables by willingness to screen with self-sampling. We used means testing for dichotomous variables by willingness to screen. We used a one-way analysis of variance (ANOVA) to compare means by physician characteristics and by influencers of women’s beliefs about avoiding pelvic exams. We used students’ t tests for comparison of means within groups of the perceptions of the exam technique. All comparisons included Bonferroni corrections for multiple comparisons. We used Statistica v. 14 [40] software.

## Results

### Population description

The collaborating health system identified and contacted 320 women who were due or overdue for cervical cancer screening. One hundred and two (102) women were interested in learning about the research study and agreed to share their contact information. Another participant referred one additional woman to the study. Of these 103 women, 20 did not respond to the initial recruitment call, 13 declined to participate, and 19 were deemed ineligible. Of the remaining 51, 40 women completed the study (20 aged 30–45 and 20 aged 46–65). Enrollment ended after 40 women completed their surveys and interviews.

### Population Characteristics

Participants self-identified as White. More than a third (15/39 (38%)) had a high school education or less, 17/39 (44%) were employed full-time, and 10/39 (26%) found it difficult or very difficult to get by on their current income. Most had health insurance (36/40 (90%)), were in good/very good/excellent health (28/39 (72%)), and had been seen by their doctor within the past 2 years (20/39 (51%)).

Table 1 provides the demographic descriptors from the quantitative survey on willingness to self-screen for cervical cancer. Willingness to screen with self-sampling was significantly higher (87.2%) than not self-screening or having no preference by all demographic descriptors (p < 0.05). The willingness to screen with self-sampling did not differ by age group, educational status, employment, insurance status, time since the last routine health check-up, income, or health status.

**Table 1:** Descriptor classification by willingness to self-test for cervical cancer screening after trialing the kits

Descriptors	I would screen with self-sampling	I would NOT screen with self-sampling	No Preference	Total
	N (Row%)	N (Row%)	N (Row%)	N (%)
Total	34 (87.2)	1 (2.6)	4 (10.3)	39 (100)
Age				
30–45 years	17 (85.0)	1 (5.0)	2 (10.0)	20 (51.3)
46–65 years	17 (89.5)	0 (0)	2 (10.5)	19 (48.7)
Education				
8-11th grade	2 (100)	0 (0)	0 (0)	2 (5.1)
High School graduate or GEDǂ	12 (92.3)	1 (7.7)	0 (0)	13 (33.3)
Vocational/technical school	2 (66.7)	0 (0)	0 (0)	3 (7.7)
Some college	7 (100)	0 (0)	0 (0)	7 (17.9)
College Graduate	10 (83.3)	0 (0)	2 (16.7)	13 (33.3)
Graduate	1 (33.3)	0 (0)	2 (66.7)	3 (7.7)
Employment				
Full time	13 (76.5)	0 (0)	4 (23.5)	17 (43.6)
Part-time	2 (100)	0 (0)	0 (0)	2 (5.1)
Student	2 (100)	0 (0)	0 (0)	2 (2.6)
Homemaker/Caretaker	5 (100)	0 (0)	0 (0)	6 (17.9)
Retired	2 (100)	0 (0)	0 (0)	2 (7.7)
Disabled	10 (90.9)	1 (9.1)	0 (0)	11 (23.1)
Insurance Status				
Employer-based	14 (77.8)	0 (0)	4 (22.2)	18 (46.2)
Purchased on own	1 (100)	0 (0)	0 (0)	1 (2.6)
Medicaid/Medicare	15 (93.8)	1 (6.3)	0 (0)	17 (43.6)
None	4 (100)	0 (0)	0 (0)	4 (10.3)
How long since your last routine health check-up				
Within past year	10 (83.3)	1 (8.3)	1 (8/3)	12 (30.8)
1–2 years	6 (75.0)	0 (0)	2 (25.0)	8 (20.5)
3–5 years	11 (91.7)	0 (0)	1 (8.3)	12 (30.8)
More than 5 years	6 (100)	0 (0)	0 (0)	6 (15.4)
Never had a routine health check-up	1 (100)	0 (0)	0 (0)	1 (2.6)
Income				
Living comfortably	13 (86.7)	0 (0)	2 (13.3)	15 (38.5)
Getting by	12 (85.7)	0 (0)	2 (14.3)	14 (35.9)
Finding it difficult to get by	8 (100)	0 (0)	0 (0)	8 (20.5)
Finding it very difficult to get by	1 (50.0)	1 (50.0)	0 (0)	2 (5.1)
Health status				
Excellent	1 (50.0)	0 (0)	1 (50.0)	2 (5.1)
Very good	12 (85.7)	1 (7.1)	1 (7.1)	14 (35.9)
Good	10 (83.3)	0 (0)	2 (16.7)	12 (30.8)
Fair	9 (100)	0 (0)	0 (0)	9 (23.1)
Poor	2 (100)	0 (0)	0 (0)	2 (5.0)

ǂGeneral educational development degree

Screening with self-sampling is significantly more preferred than not or no preference, for all descriptors, p < 0.05

#### Barriers to screening

Included four possible categories: 1) the age of the woman (demographic descriptors), 2) environmental/knowledge barriers, 3) the physician characteristics influencing women’s routine healthcare behavior, and 4) the characteristics influencing women’s participation in pelvic exams (Table 2**).** Age was not significantly associated with the preference for self-screening compared to not or no preference.

**Table 2:** Barriers to cervical cancer screening by willingness to screen by self-sampling

	I would screen with self-sampling	I would NOT screen with self-sampling	No Preference	Total
	N, row%	N, row%	N, row%	N, %
	34 (87.2)	1 (2.6)	4 (10.3)	39 (100)
Age, years	mean (SD)	mean (SD)	mean (SD)	mean (SD)
30–45 years	35.6 (4.5)	31.0 (-)	40.0 (5.7)	35.9 (4.7)
46–65 years	55.3 (6.9)	-	53.5 (4.9)	55.1 (6.6)
Total	45.9 (11.3)	31.0 (-)	46.8 (8.9)	45.6 (8.9)
Environmental/Knowledge Barriers	N, row%	N, row%	N, row%	N, %
None	11 (84.6)	0 (0)	2 (15.4)	13 (33.3)
Single	11 (91.7)	0 (0)	1 (8.3)	12 (30.8)
Uncomfortable with the pelvic exam	4 (100)	0 (0)	0 (0)	4 (10.3)
No Insurance	2 (100)	0 (0)	0 (0)	2 (5.1)
No time to get to the office	4 (80.0)	0 (0)	1 (20.0)	5 (12.8)
Uncomfortable talking about screening	1 (100)	0 (0)	0 (0)	1 (2.6)
Multiple	12 (85.7)	1 (7.1)	1 (7.1)	14 (35.9)
Physician characteristics influencing women’s health-seeking behavior for routine care*	mean (SD)	mean (SD)	mean (SD)	mean (SD)
It is important that my physician is the same gender as me for routine healthcare	3.21 (0.98)	3.0 (-)	2.75 (1.26)	3.15 (0.99)
It is important that my physician is of the same religion/culture as me for routine healthcare	3.85 (0.93)		3.75 (0.96)	3.84 (0.92)
It is important that my physician is of the same race/ethnicity as me for routine healthcare	4.18 (0.90)	4.0 (-)	4.25 (0.96)	4.18 (0.88)
Influencers of women’s pelvic exams*	mean (SD)	mean (SD)	mean (SD)	mean (SD)
I am uncomfortable/embarrassed about getting a pelvic exam	2.76 (1.46)	4.0 (-)	3.0 (0.82)	2.82 (1.39)
I have avoided a pelvic exam because of my religious/cultural beliefs,	4.32 (0.98)	2.0 (-)	4.25 (0.50)	4.26 (0.99)
I have avoided a pelvic exam because of the gender of my physician	2.85 (1.52)	4.0 (-)	2.75 (1.50)	2.87 (1.49)

^*^Scale where 1 is strongly agree—5 strongly disagree

Likewise, the absence or presence of environmental or knowledge barriers was not significantly associated with a willingness to self-screen compared to not or no preference.

On a Likert scale from 1 (strongly agree) to 5 (strongly disagree), there was no difference in the physician characteristics of gender, religion/culture, or race/ethnicity as influencers of women’s routine healthcare behavior by willingness to self-screen.

On the other hand, within influencing behaviors and among those women who prefer self-screening, the preference for a same-gender physician was significantly more important than the preference for a physician of the same religion/culture (3.21 (0.98) vs. 3.85 (0.93)) and significantly more important than the preference for a physician of the same race/ethnicity (3.21 (0.98) vs. 4.18 (0.90), ANOVA f-ratio = 10.34, p < 0.0001 and Post Hoc Tukey HSD Q_.05_ = 4.26, p < 0.001 and Q_.05_ = 6.30, p < 0.0001), respectively. Overall, the physician’s race/ethnicity was the least important, and gender was the most important factor in the willingness to self-screen.

Similarly, there was no difference in the influencers of a woman’s choice to participate in the pelvic exam by preference for self-screening. However, within the three influencers, her religion or cultural norms were significantly less likely to cause her to avoid a pelvic exam compared to her embarrassment with the pelvic exam (4.26 (0.99) vs. 2.82 (1.39)) and avoiding a pelvic exam due to the gender of the physician (2.87 (1.49) vs. 42.6 (0.99), ANOVA f-ratio = 15.54, p < 0.0001 and Post Hoc Tukey HSD Q_.05_ = 7.12, p < 0.0001 and Q_.05_ = 6.49, p < 0.0001), respectively. Overall, being uncomfortable/embarrassed or having a male physician as the reason to avoid a pelvic exam drives the willingness to self-screen.

#### Age groups

While age groups did not influence the choice to self-screen, it is important to show there were population differences in descriptors of the younger and older groups (**Supplementary Table 2**). Younger women were employed full-time more often than older women (13/17 (76.5%) vs. 4/17 (25.5%), p < 0.01), but older women self-described as disabled more often than younger women (9/11 (81.8%) vs. 2/11 (18.2%), p < 0.05). Older women had Medicaid/Medicare more often than younger women (12/17 (70.6%) vs. 5/17 (29.4%), p < 0.05), and self-reported a worse health state than younger women (9/11 (81.8%) vs. 2/11 (18.2%), p < 0.05). Finally, older women had more abortions than younger women (4/15 (26.7%) vs. 0/15 (0%), p < 0.05). Age groups were not different by education and income levels, nor by the length of time since their last health care visit. Even with these population differences, age did not contribute to the willingness to use self-sampling.

### Exam technique perceptions

**Fig. 1:**
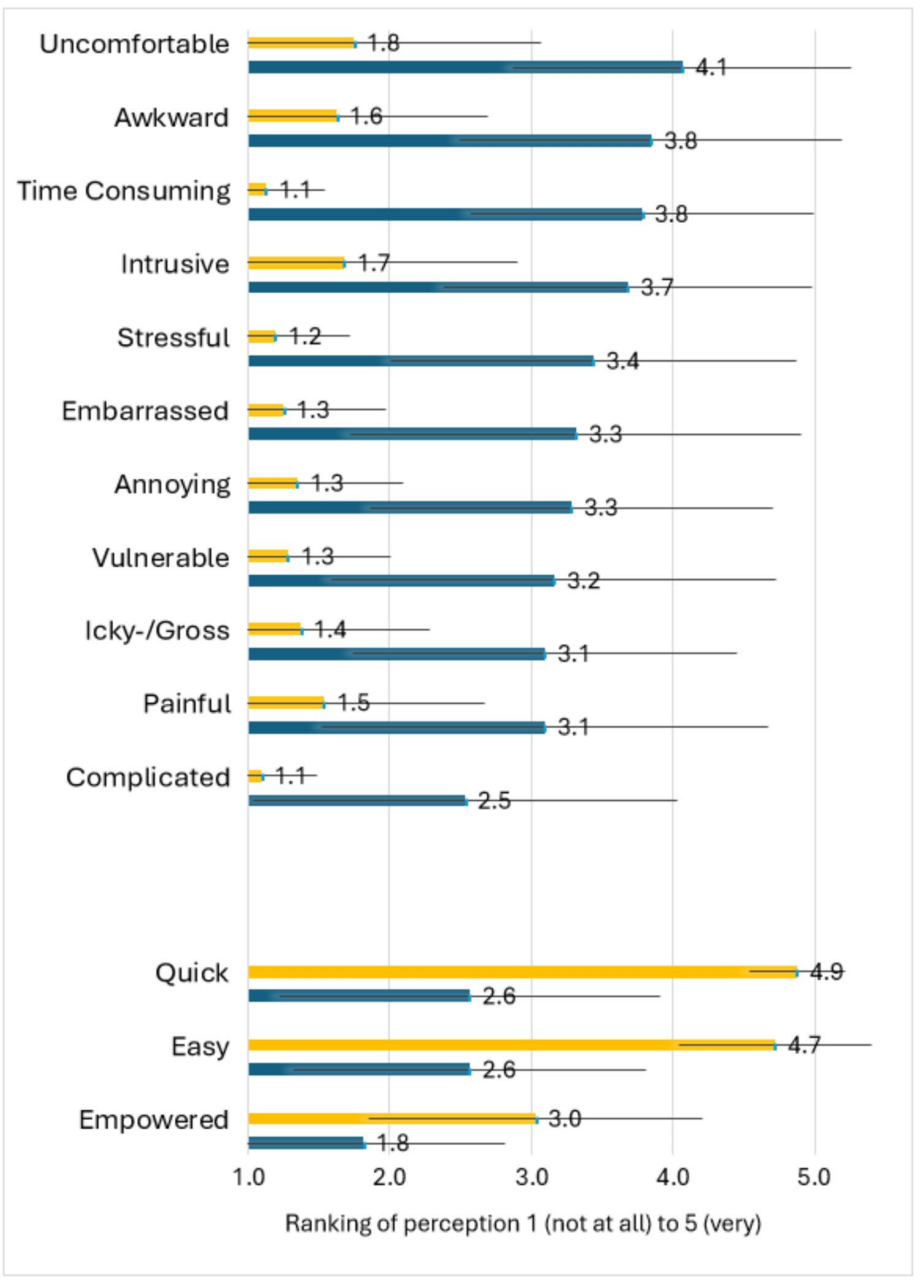
Women’s rankings of the perceptions of cervical cancer screening by exam technique, mean (SD). Yellow corresponds to “Self-sampling was” Blue corresponds to “Pelvic exam was.” The Likert scale: 1 not at all; 2 a little; 3 neutral; 4 somewhat; 5 very. Means are labeled. All pelvic exam: self-sampling technique comparisons are significantly different at p < 0.001, corrected for multiple comparisons

Comparing the women’s ratings of the perceptions of both techniques shows that self-sampling ranks significantly higher for positive perceptions and significantly lower for negative perceptions (Fig. 1).

#### Pelvic exam technique perceptions by the demographic descriptors

Supplemental Table 3 presents the six demographic descriptors by the perceptions of the pelvic exam. Only employment and income status had significant differences in perceptions of the pelvic exam. Particularly, those with full or part-time employment had worse perceptions of pelvic exam *intrusiveness* than did the retired/disabled/homemaker or student group (4.33 (1.03) vs. 3.19 (12.5), p < 0.01). Those living comfortably or getting by were minimally more *empowered* than those finding it difficult or very difficult to get by (2.06 (1.00) vs. 1.25 (0.71), p < 0.05). Supplemental Table 4 presents the six demographic descriptors by the perceptions of the *self-sampling exam*, for which there were no significant differences.

**Table 3:** Differences in Perceptions of the Pelvic exam by Environmental/ Knowledge Barriers, Physician gender, and Women’s beliefs, mean (SD)

	Environmental/Knowledge Barriers		I have avoided a pelvic exam because of the gender of my physician		I am uncomfortable/embarrassed about getting a pelvic exam	
The pelvic exam was…	None	One +	Agree	Disagree	Agree	Disagree
	N = 13	N = 26	N = 8	N = 11	N = 22	N = 12
Negative Perceptions of Pelvic Exam						
Annoying	2.69 (1.65)	3.58 (1.17)	3.39 (1.58)	2.94 (1.24)	3.77 (1.15)	2.42 (1.44)**
Uncomfortable	3.69 (1.38)	4.12 (1.14)	3.89 (1.32)	3.81 (1.22)	4.27 (0.98)	3.50 (1.51)
Intrusive	3.23 (1.59)	3.96 (1.04)	3.83 (1.25)	3.69 (1.30)	4.09 (0.87)	3.17 (1.59)*
Icky/Gross	2.38 (1.26)	3.31 (1.26)*	3.17 (1.34)	2.94 (1.18)	3.32 (1.21)	2.67 (1.44)
Embarrassing	2.54 (1.56)	3.77 (1.42)*	4.17 (1.25)	2.50 (1.32)**	3.77 (1.54)	2.58 (1.44)*
Awkward	3.15 (1.34)	4.15 (1.12)*	4.22 (1.17)	3.38 (1.20)*	4.36 (0.85)	3.00 (1.41)**
Complicated	2.23 (1.48)	2.65 (1.47)	2.67 (1.17)	2.25 (1.48)	2.64 (1.40)	2.17 (1.40)
Vulnerable	2.53 (1.67)	3.62 (1.39)*	3.78 (1.44)	2.75 (1.57)	3.73 (1.32)	2.50 (1.62)*
Stressful	2.77 (1.59)	3.73 (1.40)	3.67 (1.50)	3.00 (1.51)	3.95 (1.21)	2.67 (1.61)*
Painful	2.62 (1.71)	3.23 (1.50)	3.00 (1.53)	2.81 (1.64)	3.27 (1.55)	2.58 (1.56)
Time-Consuming	3.69 (1.38)	3.77 (1.21)	4.00 (1.08)	3.63 (1.26)	3.86 (1.28)	3.33 (1.30)
Positive Perceptions of Pelvic Exam						
Easy	3.00 (1.47)	2.27 (1.04)	2.50 (1.42)	2.69 (1.08)	2.13 (1.08)	3.17 (1.11)*
Quick	3.16 (1.68)	2.42 (1.17)	2.44 (1.34)	3.06 (1.44)	2.32 (1.25)	2.92 (1.38)
Empowering	2.54 (0.88)	1.58 (0.90)*	1.72 (0.96)	2.00 (1.03)	1.73 (0.98)	2.00 (1.04)

^*^p < 0.05, **p < 0.01

The Likert scale for ranking the perceptions of the self-sampling technique is 1 not at all, 2 a little, 3 neutral, 4 somewhat, and 5 very. Neutral (3) responses were omitted for dichotomizing the variables

For negative perception categories, the higher the number, the worse the perception. For positive perception categories, the higher the number, the better the perception

**Table 4:** Differences in Perceptions of the Self-sampling technique by physician descriptors and women’s avoidance reasons, mean (SD)

	It is important that my physician is of the same religion or culture as me for routine healthcare		It is important that my physician is of the same gender as me for routine healthcare		It is important that my physician is of the same race/ethnicity as me for routine healthcare		I have avoided a pelvic exam because of my religious and cultural beliefs		I have avoided a pelvic exam because of the gender of my physician	
Self-sampling was…	Yes N = 17	No N = 1	Yes N = 7	No N = 10	Yes N = 23	No N = 1	Yes N = 27	No N = 2	Yes N = 16	No N = 11
Negative Perceptions of Self-sampling										
Annoying	1.24 (0.75)	1.00 (0.0)	1.86 (1.07)	1.00 (0.0)*	1.30 (0.76)	1.00 (0.0)	2.00 (0.0)	1.26 (0.71)	1.44 (0.81)	1.18 (0.60)
Uncomfortable	1.36 (1.00)	1.00 (0.0)	2.14 (1.57)	1.10 (0.32)	1.65 (1.30)	1.00 (0.0)	3.00 (0.0)	1.48 (1.19)	2.25 (1.65)	1.09 (0.30)*
Intrusive	1.53 (1.18)	1.00 (0.0)	2.17 (1.60)	1.40 (0.97)	1.61 (1.12)	1.00 (0.0)	1.44 (3.00)	3.00 (0.0)	1.87 (1.19)	1.27 (0.90)
Icky/Gross	1.47 (1.01)	4.00 (0.0)*	1.86 (1.46)	1.30 (0.48)	1.35 (0.88)	4.00 (0.0)**	1.44 (0.97)	1.50 (0.71)	1.56 (1.030)	1.09 (0.30)
Embarrassing	1.18 (0.72)	1.00 (0.0)	1.71 (1.25)	1.00 (0.0)	1.26 (0.75)	1.00 (0.0)	1.15 (0.60)	2.00 (1.41)	1.38 (0.89)	1.00 (0.0)
Awkward	1.53 (1.01)	1.00 (0.0)	2.43 (1.51)	1.20 (0.42)*	1.56 (0.99)	1.00 (0.0)	3.00 (1.41)	1.56 (1.01)	2.06 (1.29)	1.18 (0.40)*
Complicated	1.06 (0.24)	1.00 (0.0)	1.43 (0.79)	1.00 (0.0)	1.04 (0.21)	1.00 (0.0)	2.00 (1.41)	1.04 (0.19)***	1.19 (0.54)	1.00 (0.0)
Vulnerable	1.18 (0.73)	1.00 (0.0)	1.86 (1.21)	1.00 (0.0)*	1.26 (0.75)	1.00 (0.0)	2.50 (0.71)	1.15 (0.60)**	1.44 (0.89)	1.00 (0.0)
Stressful	1.18 (0.53)	1.00 (0.0)	1.71 (0.95)	1.10 (0.32)	1.13 (0.46)	1.00 (0.0)	2.50 (0.71)	1.11 (0.42)***	1.31 (0.70)	1.09 (0.30)
Painful	1.47 (1.01)	1.00 (0.0)	2.29 (1.70)	1.20 (0.42)	1.52 (1.04)	1.00 (0.0)	4.00 (1.41)	1.44 (0.97)**	2.06 (1.44)	1.09 (0.30)*
Time-Consuming	1.12 (0.33)	1.00 (0.0)	1.43 (0.79)	1.10 (0.32)	1.09 (0.29)	1.00 (0.0)	2.00 (1.41)	1.07 (0.27)**	1.25 (0.58)	1.00 (0.0)
Positive Perceptions of Self-sampling										
Easy	4.82 (0.53)	5.00 (0.0)	4.43 (0.79)	5.00 (0.0)*	4.83 (0.49)	5.00 (0.0)	4.00 (0.0)	4.85 (0.46)*	4.63 (0.62)	5.00 (0.0)
Quick	4.88 (0.33)	5.00 (0.0)	4.57 (0.53)	4.90 (0.32)	4.87 (0.34)	5.00 (0.0)	4.00 (0.0)	4.89 (0.32)***	4.69 (0.48)	5.00 (0.0)*
Empowering	3.06 (1.03)	5.00 (0.0)	2.57 (0.79)	3.20 (1.14)	2.82 (1.07)	5.00 (0.0)	2.50 (0.71)	3.04 (1.16)	2.69 (1.14)	3.64 (0.81)*

^*^p < 0.05, **p < 0.01, ***p < 0.001

The Likert scale for ranking the perceptions of the self-sampling technique is 1 not at all 2 a little 3 neutral 4 somewhat 5 very. Neutral (3) responses were omitted for dichotomizing the variables to yes/no

For negative perception categories, the higher the number, the worse the perception. For positive perception categories, the higher the number, the better the perception

#### Pelvic exam technique perceptions by barrier categories

Both exam techniques were compared by each of the 14 technique perceptions, the demographic descriptors, and three barrier categories. Table 3 displays only the ratings of the significantly different perceptions of the *pelvic exam,* which are the environmental/knowledge barriers, influencers of women’s pelvic exam attendance, and physician characteristics impacting routine healthcare, on the defined five-point Likert scale (1 not at all, 2 a little, 3 neutral, 4 somewhat, and 5 very). Each of the three barriers was dichotomized. Environmental/knowledge barriers were aggregated into none vs. one or more; uncomfortable/embarrassed about getting a pelvic exam was aggregated from the five-point Likert scale to a dichotomized scale (1–strongly/agree, 2–strongly/disagree) where the neutral responses were censored. Similarly, the avoidance of a pelvic exam because of physician gender was dichotomized with the neutral responses censored.

Women with one or more environmental/knowledge barriers ranked the pelvic exam as significantly more negative for *icky/gross* (3.31 (1.26) vs. 2.38 (1.26), p < 0.05), *embarrassing* (3.77 (1.42) vs. 2.54 (1.56), p < 0.05), *awkward* (4.15 (1.12) vs. 3.15 (1.34), p < 0.05), and *makes me feel vulnerable* (3.62 (1.39) vs. 2.53 (1.67), p < 0.05) than women with no barriers. Among the positive perceptions, the pelvic exam was significantly less *empowering* for those with one or more environmental/knowledge barriers (1.58 (0.90) vs 2.54 (0.88), p < 0.05) than for those with no barriers.

Women who were uncomfortable/embarrassed about getting a pelvic exam ranked the negative perceptions of getting a pelvic exam as significantly more *annoying* (3.77 (1.15) vs. 2.42 (1.44), p < 0.01), *intrusive* (4.09 (0.87) vs. 3.17 (1.59), p < 0.05), *embarrassing* (3.77 (1.54) vs. 2.58 (1.44), p < 0.05), *awkward* (4.36 (0.85) vs. 3.00 (1.41), p < 0.01), *made them feel more vulnerable* (3.73 (1.32) vs. 2.50 (1.62), p < 0.05), and *stressful* (3.95 (1.21) vs. 2.67 (1.61), p < 0.05) than those who were not embarrassed about the pelvic exam. Among the positive perceptions, women who were uncomfortable/embarrassed about getting a pelvic exam significantly disagreed that the pelvic exam was *easy* (3.17 (1.11) vs. 2.13 (1.08), p < 0.05) with women who agreed.

Women who preferred the same-gender physician for their pelvic exam felt the pelvic exam was significantly more *embarrassing* (4.17 (1.25) vs. 2.50 (1.32), p < 0.01) and *awkward* (4.22 (1.17) vs. 3.38 (1.20), p < 0.05) than women who did not care about the gender of the physician doing the pelvic exam. All non-significant perceptions for the pelvic exam by barrier descriptions are given in Supplementary Table 5.

#### Self-sampling exam technique perceptions by barriers

Table 4 displays the significant ratings of the perceptions of the *self-sampling* technique. Unlike in Table 3, neither the number of environmental/knowledge barriers a woman had nor the impact of avoiding a pelvic exam, because it is uncomfortable/embarrassing, was associated with any perception of the self-sampling technique (Supplementary Table 6).

#### Exam technique perception by routine health-seeking behavior

For routine healthcare, women for whom their physician needed to be of their same religion/culture compared to those for whom it was not important ranked one negative perception of the self-sampling exam as significantly less *icky/gross* (1.47 (1.01) vs (4.00 (0.0), p < 0.05). All other perceptions did not differ by the physician’s religion/culture preference.

For routine healthcare, women for whom it was important to have the same-gender physician, ranked three negative perceptions of self -sampling significantly higher than those for whom having a same-gender physician was not important: *annoying* (1.86 (1.07) vs. (1.00 (0.0), p < 0.05), *awkward* (2.43 (1.51) vs. 1.29 (0.42), p < 0.05), and *made me feel vulnerable* (1.86 (1.21) vs. 1.00 (0.0), p < 0.05). All other negative perceptions of self-sampling did not differ by physician gender. While these were negative perceptions, the ranking value for each differing perception varies from "not at all" to "a little."

Among the positive perceptions of the self-sampling exam, women for whom a same-gender physician was not important had a higher ranking of *easy* (5.00 (0.0) vs. 4.43 (0.79), p < 0.05) than the women for whom a same-gender physician mattered. Similarly, regardless of preference for a same-gender physician, the positive perceptions of self-sampling, *easy* was between "very" and "somewhat."

For routine healthcare, the same race/ethnicity of the physician as the woman was significantly associated with the *icky/gross* negative perception of the self-sampling exam (1.35 (0.88) vs. 4.00 (0.0), p < 0.01).

#### Avoiding pelvic exams

A woman who avoided a pelvic exam because of her religion/culture ranked four negative perceptions of the self-sampling exam significantly worse than those women for whom her own religion/culture played no part in her getting a pelvic exam: *complicated* (2.00 (1.41) vs. 10.4 (0.19), p < 0.001), *making me feel vulnerable* (2.50 (0.71) vs. 1.15 (0.60), p < 0.01), *stressful* (2.50 (0.71) vs. 1.11 (0.42), p < 0.001), and *time-consuming* (2.00 (1.41) vs. 1.07 (0.27), p < 0.01). It must be noted, though, that the magnitude of the ranking remains at "not at all" to "a little," favoring self-sampling. The negative *painful* perception of self-sampling among women who do and do not avoid pelvic exams due to their religious/cultural beliefs is likewise significant (4.00 (1.41) "somewhat" vs. 1.44 (0.97) "not at all," p < 0.01).

Women whose religion/culture did not impact their attendance at pelvic exams were significantly more likely to rank self-sampling with two positive perceptions: *easy* (4.85 (0.46) vs. 4.00 (0.0), p < 0.05) and *quick* (4.89 (0.32) vs. 4.00 (0.0), p < 0.001), both anchored at "somewhat-very" *easy/quick*.

Having the same-gender physician causing women to avoid pelvic exams leads to significantly worse ratings of three negative perceptions for self-sampling: *uncomfortable* (2.25 (1.65) vs. 1.09 (0.30), p < 0.05), *awkward* (2.06 (1.29) vs. 1.18 (0.40), p < 0.05), *painful* (2.06 (1.44) vs. 1.09 (0.30), p < 0.05). While the difference in negative perceptions is significant, women who avoid a male physician for a pelvic exam rank self-sampling as "*a little" more uncomfortable* compared to the women who do not care about the gender of the physician, ranking self-sampling as *"not at all" uncomfortable.* This applies to all the negative perceptions.

The positive perceptions of self-sampling, *quick* (4.69 (0.48) vs. 5.0 (0.0), p < 0.05) and *empowering* (2.69 (1.14) vs. 3.64 (0.81), p < 0.05) differ by the same-gender physician causing avoidance of the pelvic exam. In this instance, those who avoid pelvic exams due to a male physician rank self-sampling as "*somewhat" quick* compared to those who do not avoid pelvic exams because of the gender of the physician, who rank self-sampling as "*very" quick*. Women who avoid pelvic exams, if they have a male physician, are "*a little" empowered* by self-sampling compared to those who do not avoid pelvic exams because of a male physician who are "*neutral-somewhat" empowered* by self-sampling.

### Qualitative and mixed-methods results

Results of the thematic analysis are presented in a joint display in Table 5 by theme, including narrative descriptions of the participants’ experiences and illustrative quotes. Participants have been assigned pseudonyms for analysis [37]. All quotes are perceptions of unique participants.

**Table 5:** A Joint Display of Qualitative and Quantitative Results

Qualitative Results		Quantitative Survey Results (1 disagree-5 agree)
Major Themes		Corresponding Perceptions from Speculum/Self technique Mean (SD)
Preference for self-sampling, either at home or in a clinic setting	"*I feel like it was a huge, huge deal to me to be able to do it at home in my own privacy when I was comfortable as opposed to going to the doctor’s office with the doctor and the assistant in the room. Like it’s completely uncomfortable for me to do it there and this made it so much easier for me. I wasn’t anxious about it. I just, you know, got it done. I didn’t have to worry*" (Ashley age 32)	Easy Speculum: 2.6 (1.2) Self: 4.7 (0.68)
Discomfort with provider-led pelvic exam	"*I got to be uncomfortable physically, and emotionally, and mentally in front of these people*" (Michelle age 45) "*I get scared and stuff when I go to the doctor and self-conscious and so I avoid having it done… I want to get screened, but I don’t feel comfortable going in. I had a doctor asked me if I was enjoying myself and that just put me right off, you know. He said it looked like I was enjoying myself and I was just staring up at the ceiling. And that made me very, very uncomfortable and, you know, it stuck with me every time I had to go in and do it.” (Deborah age 59)* "*I struggle with PTSD. I was sexually assaulted, so there is always that little bit of anxiety of going in and doing that with someone you are not 100% comfortable with. And my doctors never explained to me what they are doing. It’s like you come in, you spread your legs, they do their thing, and you leave… What are you doing and why? And why is it important? I never get any of that answered and they don’t offer that information. And a lot of women are so sick of being there for an hour anyways and you don’t want to ask the questions or you are embarrassed to ask the questions. So I think this is wonderful… I like the fact that I can do it myself. It’s less invasive and then the fact that I can do it at home is obviously a comfort."* (Amy, age 35)	Uncomfortable, Speculum:4.1 (1.2) Self:1.8 (1.3) Made me feel vulnerable, Speculum: 3.1(1.6) Self: 1.3 (0.7) Embarrassing Speculum: 3.3 (1.6) Self: 1.3 (0.7)
Self-sampling is convenient	"*You don’t have to make an appointment, you don’t have to work around your schedule, you don’t have to use your, I don’t know, lunch break or whatever to make an appointment to go do this… It’s just you and you’re at home, you mail a box and it’s incredibly simple.*" (Jenifer, age 36) "*Well, this wasn’t something I was planning on getting done anyways, and because it was so easy to do it at home, I did it… I wouldn’t have done it if I couldn’t do it at home.*" (Briana, age 37)	Quick Speculum: 2.6 (1.3) Self: 4.9 (0.3)
Self-sampling can feel empowering	"*It’s filled with confidence and low vulnerability, and hopefully, it’s effective. It will literally change women’s lives. Because think of how many more women are going to get tested because they are embarrassed to go in, so they don’t?*"	Empowering Speculum: 1.8 (1.0) Self: 3.0 (1.2)
Concerns about the accuracy of self-sampling compared to the traditional pelvic exam	"*So as long as I knew there was science to support doing it by myself was accurate, or nearly as accurate as going to the doctor, I would totally do it by myself.*" (Marie, age 37) "*I’m old school. I’m used to going to the doctor and having it sent to the lab… If it went to the same lab as my doctor, then I would definitely choose using it myself.*" (Sharon, age 56)	Complicated Speculum: 2.5 (1.5) Self: 1.1 (0.4)

Interviews provided insight into the fact that the self-sampling was *not* embarrassing, did *not* make women feel uncomfortable, and did *not* cause feelings of vulnerability compared to speculum exams

Thematic analysis of the interviews yielded five major themes, which were integrated with corresponding survey results through joint displays for a more complete understanding of preferences: 1) a preference for the self-sampling technique over the pelvic exam technique, regardless of self-sampling location (home or clinic). Specifically, all 40 women preferred self-sampling over traditional physician-directed screening when given a hypothetical choice about future screening. 2) Physical and emotional discomfort with the provider performing the pelvic exam technique. 3) Consistent negative perceptions associated with the pelvic technique; and 4) personal empowerment using the self-sampling technique. 5) Participants expressed concern about self-sampling accuracy compared to the traditional cytology or co-testing via speculum exam that they were accustomed to. As noted in Fig. 1, overall, the quantitative and qualitative results were consistent, showing ratings of the speculum exam to be worse on every perception than the self-sampling, coinciding with the person’s quote. For example, qualitative thematic results showed a preference for self-sampling and that it is convenient, and the survey results supported that self-sampling was easy and quick. In keeping with the tradition of interpretive description, in the discussion, we highlight how these findings can inform clinical care and public communication of science efforts.

## Discussion

Rural white women are rarely the prime subject of studies of cervical cancer screening disparities. While much work has been published in the Appalachian United States, the Appalachian white rural population is considered distinctly different from all other rural populations [41], making our work valuable to understanding other rural communities. Our mixed-methods study shows a very high willingness to self-sampling for cervical cancer screening, and that the perceptions of self-screening are significantly less negative and significantly more positive than for the traditional pelvic exam. Most participants found the self-sampling technique easy, quick, and empowering. By contrast, people describe the pelvic exam technique as annoying, embarrassing, and awkward, making people feel vulnerable and stressed.

We showed that rural white women who are willing to self-screen also feel that the embarrassment/uncomfortableness of the pelvic exam was the highest reason for avoiding a pelvic exam/cervical cancer screening, which the significant positive perceptions of self-sampling could alleviate. While the perceptions of self-sampling and the willingness to self-sample are greatest among women with at least one barrier to cervical cancer screening, the perceptions of and willingness to self-sample were high for all subgroups among all barriers.

We showed that rural white women with at least one knowledge or environmental barrier to screening had significantly worse perceptions of the pelvic exam, which were exacerbated by not being employed and having a difficult (very difficult) time getting by on their current income.

Likewise, we have shown that having a female physician for routine healthcare was less powerful when there was an option for self-sampling for cervical cancer screening. Similarly, if only male physicians were available for cervical cancer screening in their community, self-screening would be a highly ranked positive alternative to the pelvic exam.

Our findings demonstrate previously unappreciated barriers that rural White women face in cervical cancer screening. We are the first to show that the physician’s gender is the only characteristic that changes the under-screened, rural White woman’s negative perception of the pelvic exam technique (slightly more acceptable if the physician is female), but is immaterial to her positive perception of the self-sampling technique. In addition, we show that the physician characteristics of race/ethnicity, religion, and culture do not change these rural White women’s perceptions of the screening techniques. Moreover, we show that the woman’s own religion/culture does not change her negative perceptions of the pelvic exam technique and her positive perceptions of the self-sampling technique.

Most importantly, no social barrier changed the result that the self-sampling technique was significantly more acceptable to our rural under-screened population than the pelvic exam technique. Likewise, the women’s descriptors did not impact the collection techniques. Specifically, women from both age groups ranked the self-sampling technique more positively than the pelvic exam technique. This finding is reassuring because older women are the least likely to get screened and most likely to develop cervical cancer [1, 5, 10, 42, 43].

While others cite education level, income, insurance, and transportation as the most significant barriers to cervical cancer screening [44, 45], we sought to understand the role of the pelvic vs. self-sampling as a barrier or facilitator for screening in this rural population, finding that collection technique and setting (home vs. clinic) were salient factors for our participants. By changing to a self-sampling option, most pre-defined SEM barriers to screening disappeared.

Our research adds to the literature showing that self-sampling is a preferred option for many infections and diseases. A self-sampling option means that primary HPV testing, already proven as accurate [46], offers increased uptake in the US for screening average-risk asymptomatic women. Seventeen countries have formally adopted programmatic self-sampling for cervical cancer screening [47]. In the United States, recent changes in cervical cancer screening recommendations bring primary HPV testing to the forefront and open the door for self-sampling and home screening options [48]**.**

Lingering barriers that self-sampling cannot address are that rural White women may not know when, where, or why they are to have cervical cancer screening, may not feel comfortable discussing it with their physician, and may remain scared of abnormal results. These barriers are not specific to rural White women, though [49–51]. As physicians and researchers, we must evolve our messages about screening as the natural history and science of cervical cancer evolve. We must incorporate the option of the self-sampling technique, which can be done at home or in the office and returned by mail or in the office, which is as accurate [52], less costly [53, 54], less painful, and less inconvenient, into our messages. Specifically, teaching patients and emphasizing public health messages that self-sampling is not inferior to speculum-based testing is mandatory.

Unlike home colorectal cancer screening, which only 60% preferred [55], we anticipate self- and even home cervical cancer screening to have high utilization, similar to the high uptake of home-based comprehensive sexual health care [56, 57].

### Strengths and limitations

The unique strength of our study is our design, which prioritizes learning about women’s experiences with the techniques used in cervical cancer screening. Such investigations can help identify opportunities to increase screening rates, especially in contexts where rising rates of sexual assault [54] lead to increased risk of HPV exposure. In these contexts, having a self-sampling option for women to access screening to maximize comfort and reduce vulnerability may save lives.

Our sample size reflects a decision to purposefully recruit a smaller number of participants to learn about the experiences of white rural women **who were not up to date with their cervical cancer screening** via a combination of survey and qualitative interview responses. By providing an opportunity for open responses about the screening technique, qualitative interviews allow for the collection of richer data than surveys alone. Our interview data helped us understand *why* these women’s experiences with the self-sampling protocol differed from traditional pelvic exams and helped us gain insight into the contextual factors shaping decisions to screen. The transferability of our findings to other populations, including women in suburban and urban areas, women of different racial/ethnic backgrounds, non-binary individuals, and transgender men assigned female at birth, must be investigated in future research.

The limitations of our study include the fact that study participants had a primary care physician (PCP) assigned to them, overcoming the first barrier of finding a PCP. In addition, all recruitment occurred during the COVID pandemic, potentially making the thought of a home test more appealing. In addition, we only report on those who agreed to the survey and self-sampling, not the others who declined, creating participation bias.

The qualitative work allowed participants to voice negative and positive perceptions about the safety and effectiveness of the screening technique. The quantitative work did not focus on safety or effectiveness because prior research has shown the equivalent effectiveness of self and speculum-based sampling, and both are relatively safe [16].

In conclusion, many barriers to cervical cancer screening, including the pelvic exam technique and the physician’s gender, can be addressed with self-sampling. Self-sampling must be an option offered to anyone qualifying for cervical cancer screening.

## Supplementary Information

Below is the link to the electronic supplementary material.

Supplementary file1 (DOCX 23 KB)

Supplementary file2 (DOCX 20 KB)

Supplementary file3 (DOCX 27 KB)

Supplementary file4 (DOCX 30 KB)

Supplementary file5 (DOCX 26 KB)

## Data Availability

No datasets were generated or analyzed during the current study.
