# Supplementary material for: Rural women choose self-sampling over a pelvic exam for cervical cancer screening: a mixed-method study
Source: Cancer Causes Control. 2025 Oct 27;36(12):2023–37. doi: 10.1007/s10552-025-02081-5 (PMC12630214; doi:10.1007/s10552-025-02081-5)
Supplement: Supplementary file 1 — Supplementary file1 (DOCX 23 KB) [file 10552_2025_2081_MOESM1_ESM.docx]

Supplementary Table 1. Theoretical Domain Framework of Behavior Change Applied to Cervical Cancer screening.^24^

| Knowledge | Options for cervical cancer screening |
| --- | --- |
|  | - How long has it been since your last Pap smear? - Panel of questions about HPV infection, testing - Risk of HPV infection - Awareness of cancer, in general |
| Skills | Ability to act |
|  | - Social barriers to being screened:   - not knowing THAT screening was needed   - not knowing WHEN screening was needed   - not knowing WHERE to go for screening   - no transportation to the office   - no insurance   - uncomfortable with a pelvic exam   - uncomfortable with talking to a doctor about cervical cancer screening   - scared of knowing the results   - no time to go   - multiple inhibitory barriers |
| Social role and identity | Responsibility for their own health |
|  | - Likelihood of being screened with a vaginal self-sampling technique - Likelihood of being screened with a pelvic exam technique |
| Beliefs about capabilities | To participate in screening either via pelvic exam technique or self-sampling technique |
|  | - 14 perceptions of each of the screening techniques   - Positive Perceptions     - Quick     - Easy     - Empowering   - Negative Perceptions     - Complicated     - Time-consuming     - Stressful     - Embarrassing     - Making one feel vulnerable     - Annoying     - Icky/Gross     - Painful     - Awkward     - Intrusive     - Uncomfortable |
| Optimism/Confidence | Attaining cervical cancer prevention by completing screening |
|  | - Were you able to avoid intravaginal products for 48 hours before its use - Completion of the self-sampling kit   - How difficult was it to use   - How comfortable was it |
| Beliefs about consequences | If they do not screen |
|  | - Likelihood of developing cervical cancer |
| Reinforcement | Linking an action to cervical cancer screening |
|  | - Women's religion/culture impacts their screening participation. - Discussing options for cervical cancer screening with a physician |
| Intention to actively screen | Barriers that inhibit the intention to screen |
|  | - Importance of physician characteristics   - Importance of the gender of the physician doing the pelvic exam   - Importance of the gender of the physician for healthcare   - Importance of the religion/ethnicity of the physician for healthcare   - Importance of the race/ethnicity of the physician for healthcare - Importance of women's characteristics   - Level of discomfort/embarrassment with the pelvic exam   - Personal religion/culture leads me to avoid the pelvic exam |
| Health goals | Cervical health |
|  | - Vaccinated against HPV - Last routine healthcare exam - Last cervical cancer screening exam |
| Attention to decision processes | Brings confidence to their ability to choose the best cervical cancer sceening option for themselves |
|  | - Level of importance of positive perceptions of the screening techniques   - Quick   - Easy   - Empowering |
| Environmental context | Barriers to cervical cancer screening (such as lack of transportation), |
|  | - Social barriers   - No transportation   - No time to go |
| Social context | Who influences their decision to screen for cervical cancer |
|  | - Influence of women's religion/culture on screening |
| Emotional context | What happens during the screening experience/implication of results |
|  | - Negative perceptions of the screening techniques   - Complicated   - Time-consuming   - Stressful   - Embarrassing   - Making one feel vulnerable   - Annoying   - Icky/Gross   - Painful   - Awkward   - Intrusive   - Uncomfortable - Social barriers   - Scared of the screening results   - HPV is transmitted through the sexual act - Experience with the self-sampling kit   - Level of difficulty using the self-sampling kit   - Level of comfort using the self-sampling kit - Experience with the pelvic exam technique   - Level of difficulty with prior pelvic exam   - Level of comfort with prior pelvic exam |
| Behavioral Regulation | Confidence to carry out future plans for cervical cancer screening |
|  | - Intent to screen with self-sampling technique in the future |
