# Supplementary material for: Rural women choose self-sampling over a pelvic exam for cervical cancer screening: a mixed-method study
Source: Cancer Causes Control. 2025 Oct 27;36(12):2023–37. doi: 10.1007/s10552-025-02081-5 (PMC12630214; doi:10.1007/s10552-025-02081-5)
Supplement: Supplementary file 2 — Supplementary file2 (DOCX 20 KB) [file 10552_2025_2081_MOESM2_ESM.docx]

Supplemental Table 2. Population descriptors by age group differences

|  | Younger Age | Older Age | Total |  |
| --- | --- | --- | --- | --- |
| **Education** | **N, Row%** | **N** | **N, %** | **p-value** |
| 8-11th grade | 0 (0) | 2 (100) | 2 (5.0) | NS |
| HS graduate or GED | 5 (38.5) | 8 (61.5) | 13 (32.5) |  |
| Vocational/technical school | 2 (66.7) | 1 (33.3) | 3 (7.5) |  |
| Some college | 4 (57.1) | 3 (42.9) | 7 (17.5) |  |
| College Graduate | 8 (66.7) | 4 (33.3) | 12 (30.0) |  |
| Graduate | 1 (33.3) | 2 (66.7) | 3 (7.5) |  |
| **Income** |  |  |  | NS |
| Living comfortably | 7 (46.7) | 8 (53.3) | 15 (37.5) |  |
| Getting by | 8 (53.3) | 7 (46.7) | 15 (37.5) |  |
| Finding it difficult to get by | 4 (50.0) | 4 (50.0) | 8 (20.0) |  |
| Finding it very difficult to get by | 1 (50.0) | 1 (50.0) | 2 (5.0) |  |
| **How long since your last routine health check-up** | | |  | NS |
| Within past year | 9 (75.0) | 3 (25.0) | 12 (30.0) |  |
| 1-2 years | 3 (37.5) | 5 (62.5) | 8 (20.0) |  |
| 3-5 years | 6 (46.2) | 7 (53.79 | 13 (32.5) |  |
| More than 5 years | 1 (16.7) | 5 (83.6) | 6 (15.0) |  |
| Never had a routine health check-up | 1 (100) | 0 (0.0) | 1 (2.5) |  |
| **Employment** |  |  |  |  |
| Full time | 13 (76.5) | 4 (23.5) | 17 (42.5) | **<0.01** |
| Part-time | 0 (0.0) | 2 (100) | 2 (5.0) |  |
| Student | 1 (50.0) | 1 (50.0) | 2 (5.0) |  |
| Homemaker/Caretaker | 4 (66.7) | 2 (33.3) | 6 (15.0) |  |
| Retired | 0 (0.0) | 2 (100) | 2 (5.0) |  |
| Disabled | 2 (18.2) | 9 (81.8) | 11 (27.5) | **<0.05** |
| **Insurance Status** |  |  |  |  |
| Employer-based | 11 (61.1) | 7 (38.9) | 18 (45.0) |  |
| Purchased on own | 1 (100) | 0 (0.0) | 1 (2.5) |  |
| Medicaid/Medicare | 5 (29.4) | 12 (70.6) | 17 (42.5) | **<0.05** |
| None | 3 (75.0) | 1 (25.0) | 4 (10.0) |  |
| **Health status*** |  |  |  |  |
| Excellent | 1 (50.0) | 1 (50.0) | 2 (5.0) |  |
| Very good | 9 (64.3) | 5 (35.7) | 14 (35.0) |  |
| Good | 8 (61.5) | 5 (38.5) | 13 (32.5) |  |
| Fair | 1 (11.1) | 8 (88.9) | 9 (22.5) | **<0.05** |
| Poor | 1 (50.0) | 1 (50.0) | 2 (5.0) |  |
| **Birth History** |  |  |  |  |
| Number of times pregnant | 17 (47.2) | 19 (52.8) | 36 (100) |  |
| Number of deliveries | 16 (45.7) | 19 (54.3) | 35 (100) |  |
| Number of abortions | 0 (0.0) | 4 (100) | 4 (100) | **<0.05** |

*Aggregating excellent/very good/good and fair and poor. Younger people are in significantly better health than older people, p<0.05
