# Supplementary material for: Rural women choose self-sampling over a pelvic exam for cervical cancer screening: a mixed-method study
Source: Cancer Causes Control. 2025 Oct 27;36(12):2023–37. doi: 10.1007/s10552-025-02081-5 (PMC12630214; doi:10.1007/s10552-025-02081-5)
Supplement: Supplementary file 3 — Supplementary file3 (DOCX 27 KB) [file 10552_2025_2081_MOESM3_ESM.docx]

Supplementary Table 3. Demographic Descriptors by the Perception of the Pelvic Exam

|  | **Employment** | | **Income** | | | **Insurance** | | **Age** | | |
| --- | --- | --- | --- | --- | --- | --- | --- | --- | --- | --- |
|  | Full/parttime | Retired/disabled/ homemaker/stdnt | Comfortably/ getting by | Finding it very/difficult | | Employer/Self | Federal/ State | 30-45 y | 46-65 y | |
|  | N=18 | N=21 | N=31 | N=8 | | N=18 | N=17 | N=19 | N=20 | |
| ANNOYING | 3.28 (1.36) | 3.29 (1.45) | 3.19 (1.42) | 3.63 (1.30) | | 3.22 (1.35) | 3.35 (1.46) | 3.26 (1.33) | 3.30 (1.49) | |
| EASY | 2.67 (1.41) | 2.38 (1.07 | 2.55 (1.34) | 2.38 (0.74) | | 2.44 (1.29) | 2.53 (1.18) | 2.58 (1.35) | 2.45 (1.15) | |
| INTRUSIVE | **4.33 (1.03)** | **3.19 (1.25)**** | 3.81 (1.25) | 3.38 (1.41) | | 4.00 (1.19 ) | 3.41 (1.23) | 3.89 (1.24) | 3.55 (1.32) | |
| QUICK | 2.72 (1.45) | 2.62 (1.36) | 2.71 (1.42) | 2.50 (1.31) | | 2.78 (1.44) | 2.71 (1.36) | 2.68 (1.38) | 2.65 (1.42) | |
| ICKY or GROSS | 3.33 (1.14) | 2.71 (1.42) | 3.00 (1.24) | 3.00 (1.69) | | 3.00 (1.08) | 2.82 (1.42) | 3.37 (1.42) | 2.65 (1.14) | |
| EMBARRASSING | 3.44 (1.54) | 3.29 (1.62) | 3.23 (1.56) | 3.88 (1.55) | | 2.94 (1.59) | 3.71 (1.45) | 3.32 (1.60) | 3.40 (1.57) | |
| EMPOWERING | 2.06 (1.00) | 1.76 (1.00) | **2.06 (1.00)** | **1.25 (0.71)*** | | 2.06 (1.00) | 1.94 (1.03) | 1.79 (0.98) | 2.00 (1.03) | |
| UNCOMFORTABLE | 3.89 (1.28) | 4.05 (1.20) | 3.90 (0.27) | 4.25 (1.04) | | 4.00 (1.14) | 4.06 (1.20) | 3.68 (1.38) | 4.25 (1.020) | |
| AWKWARD | 4.06 (1.16) | 3.62 (1.36) | 3.84 (1.24 ) | 3.75 (1.49) | | 3.56 (1.34) | 4.00 (1.17) | 3.79 (1.47) | 3.85 (1.09) | |
| COMPLICATED | 2.61 (1.46) | 2.43 (1.50) | 2.45 (1.41) | 2.75 (1.75) | | 2.50 (1.42) | 2.53 (1.55) | 2.37 (1.50) | 2.65 (1.46) | |
| My last pelvic exam made me feel VULNERABLE | 3.72 (1.18) | 2.86 (1.74) | 3.06 (1.50) | 4.00 (1.60) | | 3.17 (1.47) | 3.29 (1.65) | 3.37 (1.50) | 3.15 (1.63) | |
| STRESSFUL | 3.28 (1.45) | 3.52 (1.60) | 3.32 (1.45) | 3.75 (1.83) | | 3.06 (1.39) | 3.71 (1.57) | 2.95 (1.61) | 3.85 (1.31) | |
| PAINFUL | 3.22 (1.63) | 2.86 (1.56) | 2.97 (1.62) | 3.25 (1.49) | | 3.17 (1.65) | 2.94 (1.56) | 2.74 (1.59) | 3.30 (1.56) | |
| TIME-CONSUMING | 3.94 (1.00) | 3.57 (1.43) | 3.77 (1.15) | 3.63 (1.69) | | 4.00 (1.03) | 3.53 (1.37) | 3.95 (1.08) | 3.55 (1.39) | |
|  | **p<0.01 |  | *p<0.05 |  | | NS |  | NS |  | |
|  | **Education** | | **Routine check-up** | | **Health Status** | | |  | |  |
|  | HS or less | More than HS | 2 or less yrs | more than 2 y | | Excellent/  very/good | Fair/poor |  |  | |
|  | N=15 | N=24 | N=20 | N=17 | | N=28 | N=11 |  |  | |
| ANNOYING | 3.27 (1.16) | 3.29 (1.55) | 3.05 (1.64) | 3.47 (1.07) | | 3.07 (1.36) | 3.82 (1.40) |  |  | |
| EASY | 2.60 (0.91) | 2.46 (1.41) | 2.65 (1.31) | 2.53 (1.12) | | 2.57 (1.29) | 2.36 (1.12) |  |  | |
| INTRUSIVE | 3.40 (0.83) | 3.92 (1.47) | 3.45 (1.39) | 3.88 (1.11) | | 3.93 (1.18) | 3.18 (1.40) |  |  | |
| QUICK | 2.80 (1.26) | 2.58 (1.47) | 2.80 (1.47) | 2.65 (1.32) | | 2.75 (1.32) | 2.45 (1.57) |  |  | |
| ICKY or GROSS | 2.93 (0.88) | 3.04 (1.55) | 3.10 (1.48) | 2.82 (1.19) | | 3.04 (1.23) | 2.91 (1.58) |  |  | |
| EMBARRASSING | 3.27 (1.49) | 3.42 (1.64) | 3.10 (1.62) | 3.47 (1.50) | | 3.14 (1.53) | 3.91 (1.58) |  |  | |
| EMPOWERING | 2.07 (1.03) | 1.79 (0.98) | 1.80 (1.01) | 2.00 (1.00) | | 1.96 (1.00) | 1.73 (1.01) |  |  | |
| UNCOMFORTABLE | 4.07 (0.96) | 3.92 (1.38) | 3.65 (1.42) | 4.29 (0.92) | | 3.86 (1.21) | 4.27 (1.27) |  |  | |
| AWKWARD | 3.73 (1.22) | 3.88 (1.33) | 3.55 (1.36) | 4.12 (1.17) | | 3.79 (1.26) | 3.91 (1.38) |  |  | |
| COMPLICATED | 2.33 (1.11) | 2.63 (1.66) | 2.30 (1.42) | 2.71 (1.61) | | 2.46 (1.35) | 2.64 (1.80) |  |  | |
| My last pelvic exam made me feel VULNERABLE | 2.67 (1.45) | 3.63 (1.53) | 3.05 (1.64) | 3.29 (1.45) | | 3.29 (1.46) | 3.18 (1.83) |  |  | |
| STRESSFUL | 3.13 (1.30) | 3.58 (1.64) | 3.20 (1.54) | 3.47 (1.50) | | 3.18 (1.47) | 4.00 (1.55) |  |  | |
| PAINFUL | 3.20 (1.52) | 2.92 (1.64) | 2.75 (1.62) | 3.53 (1.46) | | 2.89 (1.59) | 3.36 (1.57) |  |  | |
| TIME-CONSUMING | 3.53 (1.13) | 3.88 (1.33) | 3.90 (1.21) | 3.59 (1.28) | | 3.79 (1.10) | 3.64 (1.63) |  |  | |
|  | NS |  | NS |  | | NS |  |  |  | |
