# Supplementary material for: Rural women choose self-sampling over a pelvic exam for cervical cancer screening: a mixed-method study
Source: Cancer Causes Control. 2025 Oct 27;36(12):2023–37. doi: 10.1007/s10552-025-02081-5 (PMC12630214; doi:10.1007/s10552-025-02081-5)
Supplement: Supplementary file 4 — Supplementary file4 (DOCX 30 KB) [file 10552_2025_2081_MOESM4_ESM.docx]

Supplementary Table 4. Nonsignificant differences of the Demographic Descriptors bythe Perceptions of Self-Sampling

|  | **Education** | | | **Employment** | | **Income** | | | **Age** | | |
| --- | --- | --- | --- | --- | --- | --- | --- | --- | --- | --- | --- |
|  | HS or less | More than HS | | Full/parttime | Retired/Disable/Homemaker/Student | Comfortable/  getting by | | Finding it very/difficult | 30-45 y | | 46-65 y |
|  | N=11 | N=21 | | N=16 | N=16 | N=25 | | N=7 | N=18 | | N=14 |
| ANNOYING to use | 1.36 (0.67) | 1.33 (0.80) | | 1.56 (0.96) | 1.13 (0.34) | 1.40 (0.81) | | 1.14 (0.38) | 1.33 (0.84) | | 1.36 (0.63) |
| EASY to use | 4.82 (0.40) | 4.81 (0.51) | | 4.81 (0.54) | 4.81 (0.40) | 4.76 (0.53) | | 5.00 (0.0) | 4.78 (0.55) | | 4.86 (0.36) |
| INTRUSIVE to use | 1.50 (0.85) | 1.62 (1.16) | | 1.75 (1.29) | 1.40 (0.74) | 1.54 (1.06) | | 1.71 (1.11) | 1.72 (1.23) | | 1.38 (0.36) |
| QUICK to use | 4.82 (0.40) | 4.86 (0.36) | | 4.81 (0.40) | 4.88 (0.34) | 4.80 (0.41) | | 5.00 (0.0) | 4.83 (0.38) | | 4.86 (0.36) |
| ICKY or GROSS to use | 1.09 (1.19) | 1.57 (1.08) | | 1.56 (1.03) | 1.25 (0.77) | 1.36 (0.86) | | 1.57 (1.13) | 1.61 (1.14) | | 1.14 (0.36) |
| EMBARRASSING to use | 1.45 (0.82) | 1.14 (0.65) | | 1.31 (0.87) | 1.19 (0.55) | 1.28 (0.79) | | 1.14 (0.38) | 1.28 (0.83) | | 1.21 (0.58) |
| EMPOWERING to use | 2.73 (1.19) | 3.24 (1.09) | | 3.25 (0.77) | 2.88 (1.41) | 3.08 (1.04) | | 3.00 (1.53) | 3.33 (0.97) | | 2.71 (1.27) |
| UNCOMFORTABLE to use | 1.82 (1.17) | 1.67 (1.43) | | 1.69 (1.40) | 1.75 (1.29) | 1.80 (1.38) | | 1.43 (1.13) | 1.61 (1.33) | | 1.86 (1.35) |
| AWKWARD to use | 1.55 (1.04) | 1.71 (1.10) | | 2.00 (1.21) | 1.31 (0.79) | 1.72 (1.06) | | 1.43 (1.13) | 1.72 (1.07) | | 1.57 (1.09) |
| COMPLICATED to use | 1.18 (0.60) | 1.05 (0.22) | | 1.06 (0.25) | 1.13 (0.50) | 1.12 (0.44) | | 1.00 (0.0) | 1.06 (0.24) | | 1.14 (0.53) |
| Made me feel VULNERABLE | 1.45 (0.82) | 1.19 (0.68) | | 1.38 (0.89) | 1.19 (0.54) | 1.36 (0.81) | | 1.00 (0.0) | 1.33 (0.84) | | 1.21 (0.58) |
| STRESSFUL to use | 1.27 (0.65) | 1.14 (0.48) | | 1.19 (0.54) | 1.19 (0.54) | 1.24 (0.60) | | 1.00 (0.0) | 1.11 (0.47) | | 1.29 (0.61) |
| PAINFUL to use | 1.64 (1.29) | 1.52 (1.08) | | 1.50 (1.03) | 1.63 (1.26) | 1.64 (1.25) | | 1.29 (0.49) | 1.61 (1.14) | | 1.50 (1.16) |
| TIME-CONSUMING to use | 1.18 (0.60) | 1.10 (0.30) | | 1.13 (0.34) | 1.13 (0.50) | 1.16 (0.47) | | 1.00 (0.0) | 1.11 (0.32) | | 1.14 (0.53) |
|  | **Insurance** | | **Routine check-up** | | | | **Health Status** | |  |  | |
|  | Employer/Self | Federal/State | | 2 or less yrs | More than 2 yrs | Excellent/  very/good | | Fair/poor |  | |  |
|  | N=16 | N=13 | | N=16 | N=14 | N=24 | | N=8 |  | |  |
| ANNOYING to use | 1.50 (0.97) | 1.23 (0.44) | | 1.25 (0.58) | 1.50 (0.94) | 1.33 (0.82) | | 1.38 (0.52) |  | |  |
| EASY to use | 4.81 (0.54) | 4.77 (0.44) | | 4.81 (0.40) | 4.79 (0.58) | 4.88 (0.45) | | 4.63 (0.52) |  | |  |
| INTRUSIVE to use | 1.56 (1.15) | 1.50 (0.80) | | 1.20 (0.56) | 1.86 (1.35) | 1.67 (1.13) | | 1.29 (0.76) |  | |  |
| QUICK to use | 4.81 (0.40) | 4.85 (0.38) | | 4.88 (0.34) | 4.86 (0.36) | 4.88 (0.34) | | 4.75 (0.46) |  | |  |
| ICKY or GROSS to use | 1.50 (1.03) | 1.38 (0.87) | | 1.38 (0.81) | 1.43 (1.09) | 1.33 (0.87) | | 1.63 (1.06) |  | |  |
| EMBARRASSING to use | 1.31 (0.87) | 1.23 (0.60) | | 1.13 (0.50) | 1.43 (0.94) | 1.25 (0.74) | | 1.25 (0.71) |  | |  |
| EMPOWERING to use | 3.25 (1.06) | 2.77 (1.30) | | 3.19 (1.11) | 2.86 (1.10) | 3.13 (1.15) | | 2.88 (1.13) |  | |  |
| UNCOMFORTABLE to use | 1.69 (1.40) | 1.92 (1.38) | | 1.50 (1.10) | 1.79 (1.37) | 1.75 (1.45) | | 1.63 (0.92) |  | |  |
| AWKWARD to use | 1.81 (1.10) | 1.62 (1.12) | | 1.69 (1.08) | 1.64 (1.15) | 1.54 (0.98) | | 2.00 (1.31) |  | |  |
| COMPLICATED to use | 1.06 (0.25) | 1.15 (0.55) | | 1.00 (0.0) | 1.21 (0.58) | 1.04 (0.20) | | 1.25 (0.71) |  | |  |
| Made me feel VULNERABLE | 1.38 (0.89) | 1.23 (0.60) | | 1.13 (0.34) | 1.50 (1.02) | 1.25 (0.74) | | 1.38 (0.74) |  | |  |
| STRESSFUL to use | 1.19 (0.54) | 1.23 (0.60) | | 1.13 (0.34) | 1.29 (0.73) | 1.13 (0.45) | | 1.38 (0.74) |  | |  |
| PAINFUL to use | 1.44 (1.03) | 1.77 (1.36) | | 1.63 (1.36) | 1.50 (0.94) | 1.38 (0.88) | | 2.13 (1.64) |  | |  |
| TIME-CONSUMING to use | 1.13 (0.34) | 1.15 (0.55) | | 1.19 (0.54) | 1.07 (0.27) | 1.08 (0.28) | | 1.25 (0.71) |  | |  |
