# Supplementary material for: Rural women choose self-sampling over a pelvic exam for cervical cancer screening: a mixed-method study
Source: Cancer Causes Control. 2025 Oct 27;36(12):2023–37. doi: 10.1007/s10552-025-02081-5 (PMC12630214; doi:10.1007/s10552-025-02081-5)
Supplement: Supplementary file 5 — Supplementary file5 (DOCX 26 KB) [file 10552_2025_2081_MOESM5_ESM.docx]

Supplementary Table 5. Non-significant perceptions of the Pelvic Exam technique by personal beliefs and characteristics of the physician providing routine health care

|  | I have avoided a pelvic exam because of my religious/cultural beliefs. | | It is important that my physician be the same gender as me for routine healthcare. | | It is important that my physician be of the same religion/culture as me for routine healthcare. | | It is important that my physician be of the same race/ethnicity as me for routine healthcare. | |
| --- | --- | --- | --- | --- | --- | --- | --- | --- |
| **Negative Perceptions** | Agree  N=3 | Disagree  N=33 | Agree  N=8 | Disagree  N=11 | Agree  N=1 | Disagree  N=20 | Agree  N=1 | Disagree  N=29 |
| Annoying | 4.00 (1.00) | 3.21 (1.47) | 3.63 (1.51) | 3.00 (1.26) | 5.00 (-) | 3.15 (0.39) | 5.00 (-) | 3.28 (1.39) |
| Intrusive | 3.67 (0.58) | 3.70 (1.36) | 3.88 (1.25) | 4.09 (0.83) | 5.00 (-) | 3.90 (1.21) | 5.00 (-) | 3.66 (1.29) |
| Icky/Gross | 3.00 (1.00) | 3.06 (1.34) | 3.00 (1.20) | 3.63 (0.92) | 5.00 (-) | 3.35 (1.18) | 5.00 (-) | 2.97 (1.27) |
| Embarrassing | 4.00 (1.00) | 3.27 (1.57) | 4.25 (1.04) | 3.18 (1.47) | 5.00 (-) | 3.4 (1.60) | 5.00 (-) | 3.24 (1.57) |
| Uncomfortable | 4.33 (1.15) | 3.85 (1.25) | 4.13 (1.13) | 3.91 (1.14) | 5.00 (-) | 3.70 (1.30) | 5.00 (-) | 3.86 (1.19) |
| Awkward | 4.33 (1.15) | 3.70 (1.31) | 4.13 (1.13) | 3.82 (1.08) | 5.00 (-) | 3.65 (1.39) | 5.00 (-) | 3.69 (1.34) |
| Complicated | 2.33 (1.15) | 2.55 (1.54) | 2.63 (1.69) | 2.27 (1.27) | 4.00 (-) | 2.50 (1.50) | 4.00 (-) | 2.62 (1.52) |
| made me feel Vulnerable | 3.33 (0.58) | 3.18 (1.65) | 3.38 (1.41) | 3.09 (1.58) | 5.00 (-) | 3.25 (1.62) | 5.00 (-) | 3.14 (1.57) |
| Stressful | 3.67 (0.58) | 3.33 (1.57) | 3.88 (1.25) | 3.45 (1.51) | 5.00 (-) | 3.25 (1.68) | 5.00 (-) | 3.28 (1.56) |
| Painful | 4.00 (1.00) | 2.91 (1.59) | 3.38 (1.60) | 2.72 (1.68) | 4.00 (-) | 2.75 (1.59) | 4.00 (-) | 2.93 (1.56) |
| Time-Consuming | 4.00 (1.00) | 3.88 (1.19) | 4.38 (0.52) | 3.55 (1.21) | 5.00 (-) | 3.75 (1.21) | 5.00 (-) | 3.83 (1.10) |
| **Positive Perceptions** |  |  |  |  |  |  |  |  |
| Easy | 2.33 (0.58) | 2.58 (1.30) | 2.25 (1.16) | 2.82 (1.17) | 1.00 (-) | 2.65 (1.27) | 1.00 (-) | 2.62 (1.29) |
| Quick | 2.00(1.00) | 2.76 (1.46) | 2.13 (1.13) | 3.00 (1.34) | 1.00 (-) | 2.75 (1.29) | 1.00 (-) | 2.79 (1.40) |
| Empowering | 2.33 (1.15) | 1.82 (0.98) | 2.00 (1.07) | 1.91 (1.04) | 1.00 (-) | 1.85 (0.99) | 1.00 (-) | 1.93 (1.00) |

The Likert scale for ranking the perceptions of the pelvic exam technique is 1 not at all, 2 a little, 3 neutral, 4 somewhat, and 5 very. The neutral rankings were omitted when dichotomizing the variables to agree/disagree.

The perceptions of the pelvic exam did not differ by women’s religious/cultural beliefs, by a physician for her routine health care who was male, of the same religion/culture as she, or the same race/ethnicity.
